# Association of *APOE ε4/ε4* with fluid biomarkers in patients from the PUMCH dementia cohort

**DOI:** 10.3389/fnagi.2023.1119070

**Published:** 2023-03-31

**Authors:** Li Shang, Liling Dong, Xinying Huang, Tianyi Wang, Chenhui Mao, Jie Li, Jie Wang, Caiyan Liu, Jing Gao

**Affiliations:** Neurological Department, State Key Laboratory of Complex Severe and Rare Disease, Peking Union Medical College Hospital, Chinese Academy of Medical Science and Peking Union Medical College, Beijing, China

**Keywords:** *APOE ε4/ε4*, CSF biomarker, plasma biomarker, dementia, Alzheimer’s disease

## Abstract

**Background:**

Apolipoprotein-E (*APOE*) ε4 is a major genetic risk factor for Alzheimer’s disease (AD). Current studies, which were mainly based on the clinical diagnosis rather than biomarkers, come to inconsistent conclusions regarding the associations of *APOE ε4* homozygotes (*APOE ε4/ε4*) and cerebrospinal fluid (CSF) biomarkers of AD. In addition, few studies have explored the associations of *APOE ε4/ε4* with plasma biomarkers. Therefore, we aimed to investigate the associations of *APOE ε4/ε4* with fluid biomarkers in dementia and biomarker-diagnosed AD.

**Methods:**

A total of 297 patients were enrolled. They were classified into Alzheimer’s continuum, AD, and non-AD, according to CSF biomarkers and/or β amyloid PET results. AD was a subgroup of the AD continuum. Plasma Amyloid β (Aβ) 40, Aβ42, glial fibrillary acidic protein (GFAP), neurofilament light chain (NFL), and phosphorylated tau (P-tau)181 were quantified in 144 of the total population using an ultra-sensitive Simoa technology. We analyzed the associations of *APOE ε4/ε4* on CSF and plasma biomarkers in dementia and biomarker diagnosed AD.

**Results:**

Based on the biomarker diagnostic criteria, 169 participants were diagnosed with Alzheimer’s continuum and 128 individuals with non-AD, and among the former, 120 patients with AD. The *APOE ε4/ε4* frequencies were 11.8% (20/169), 14.2% (17/120), and 0.8% (1/128) in Alzheimer’s continuum, AD and non-AD, respectively. Only CSF Aβ42 was shown to be decreased in *APOE ε4/ε4* carriers than in non-carriers for patients with AD (*p* = 0.024). Furthermore, we did not find any associations of *APOE ε4* with plasma biomarkers of AD and non-AD. Interestingly, we found that in non-AD patients, *APOE ε4* carriers had lower CSF Aβ42 (*p* = 0.018) and higher T-tau/Aβ42 ratios (*p* < 0.001) and P-tau181/Aβ42 ratios (*p* = 0.002) than non-carriers.

**Conclusion:**

Our data confirmed that of the three groups (AD continuum, AD, and non-AD), those with AD had the highest frequency of *APOE ɛ4/ɛ4* genotypes. The *APOE ɛ4/ɛ4* was associated with CSF levels of Aβ42 but not tau for AD and non-AD, suggesting that *APOE ɛ4/ɛ4* affected the Aβ metabolism of both. No associations between *APOE ε4/ɛ4* and plasma biomarkers of AD and non-AD were found.

## 1. Introduction

Alzheimer’s disease (AD) is the leading cause of dementia in elderly individuals. Its characteristic pathological changes are the extracellular deposits of Aβ protein and the intracellular accumulation of phosphorylated tau protein (Yamazaki et al., 2019). The apolipoprotein-E (*APOE*) *ε4* allele is the strongest genetic risk factor for AD (Corder et al., 1993). In addition, the *APOE ε4* also affects the risk for other dementias, such as vascular dementia (VAD; Rohn, 2014), frontotemporal lobar degeneration (FTLD), and Lewy body disease (LBD; Belloy et al., 2019). In humans, the gene exists in three allele variants called *ε2*, *ε3*, and *ε4*. In comparison to the *APOE ε3/ε3*, a single copy of the *APOE ε4* allele results in a 3- to 4-fold increase in the risk for AD, and *APOE ε4/ε4* results in a 9- to 15-fold increase (Liu et al., 2013; Yamazaki et al., 2019).

Numerous published studies have focused on the *APOE ε4* and AD pathological changes. The correlation between *APOE ε4* and AD cerebrospinal fluid (CSF) biomarkers was explored by most studies (Lautner et al., 2014; Mattsson et al., 2018; Bussy et al., 2019; Konijnenberg et al., 2020; Benson et al., 2022). However, their results were inconsistent. The inclusion of AD patients based on clinical diagnosis alone may be the cause. While according to the 2018 National Institute on Aging Alzheimer’s Association (NIA-AA) research framework (Jack et al., 2018), we can make a diagnosis based on biomarkers. The biomarker diagnosis was more sensitive and specific for the AD neuropathologic changes relative to the clinical diagnosis (Jack et al., 2018; Saddiki et al., 2020). However, there were relatively few studies on the associations between *APOE ε4* and CSF biomarkers in biomarker-diagnosed AD. Moreover, due to the low carriage rate of *APOE ε4/ε4* in the population, most studies have only dichotomized the included subjects based on whether they carry the *APOE ε4*, which also seems to obscure the uniqueness of *APOE ε4/ε4*.

Moreover, plasma biomarker testing with low invasiveness and low cost for AD showed promise (Teunissen et al., 2022). Limited studies have demonstrated that the number of *APOE ε4* alleles was not associated with plasma Amyloid β (Aβ) 40, Aβ42, Aβ42/Aβ40, and phosphorylated tau (P-tau)181 for AD patients (Janelidze et al., 2016; Salami et al., 2022). Glial fibrillary acidic protein (GFAP), a reactive astrogliosis biomarker, is a promising candidate biomarker for AD (Pereira et al., 2021). Similarly, the neurofilament light chain (NFL) is a sensitive biomarker for neuroaxonal damage. Plasma levels of NFL are correlated with future atrophy, hypometabolism, and cognitive decline for AD (Mattsson et al., 2019). However, few studies have examined the associations of *APOE ε4* with plasma GFAP and NFL for AD.

In the present study, we aimed to investigate the associations of *APOE ɛ4/ε4* with both CSF and plasma biomarkers in *dementia and biomarker diagnosed AD*. We expected to gain a deeper understanding of the impact of *APOE ɛ4/ε4* on AD pathology.

## 2. Methods

### 2.1. Participants

We used data from the Peking Union Medical College Hospital (PUMCH) dementia cohort. The study received approval from the ethics committee of the PUMCH and was conducted in compliance with the Declaration of Helsinki. Written informed consent was obtained from all subjects.

A total of 297 patients with dementia were enrolled. The inclusion criteria were as follows: 1. All patients met the diagnostic criteria for all-cause dementia as defined by the NIA-AA (McKhann et al., 2011). 2. All patients underwent the history inquiry, neurological examination, blood biochemical test (i.e., hepatic function, renal function, homocysteine, thyroid function, folic acid, vitamin B12, blood ammonia, and rapid plasma reagin test), neuroimaging and neuropsychological assessment, CSF testing, and *APOE* genotyping. The exclusion criteria were as follows: 1. Patients diagnosed with dementia caused by acquired etiologies (e.g., infectious, toxic, metabolic, and neoplastic diseases). 2. Patients diagnosed with undetermined dementia.

All included patients completed a neuropsychological assessment, CSF biomarker testing, and *APOE* genotyping. Of these, 144 patients finished the plasma biomarkers testing.

### 2.2. Neuropsychological assessment

A step-by-step cognitive assessment system developed by our laboratory was used, including cognitive screening and cognitive composite. The cognitive screening included a mini-mental state examination (MMSE), Montreal cognitive assessment (PUMCH edition; Tan et al., 2015), activities of daily living (ADL), and hospital anxiety and depression scale (HAD). The cognitive composite consisted of more than 20 neuropsychological subtests that assessed five cognitive domains, including executive function, visuospatial function, language function, memory function (verbal and nonverbal memory), and conceptual reasoning and computation. This has been explained in detail previously (Wang et al., 2022).

### 2.3. CSF biomarkers

All participants underwent lumbar CSF sampling. Samples were stored in a low protein binding tube and centrifuged at 1,800*g* for 10 min at 4°C within 24 h after collection. The supernatant was transferred to a new tube and stored at −80°C. Commercial accessible ELISA kits were used for the analysis of CSF T-tau, P-tau181, and Aβ42 with INNOTEST hTAU Ag, PHOSPHO-TAU, and β-AMYLOID (1-42) (Fujirebio, Ghent, Belgium). All analyses were performed by board-certified laboratory technicians, who were blinded to clinical data and diagnoses.

### 2.4. Plasma biomarkers

Blood samples collected in *EDTA* tubes were centrifuged at 3,500 rpm for 15 min and plasma was removed. Then, the plasma samples were *frozen* at −80°C and were freeze-thawed only once. EDTA plasma Aβ40, Aβ42, GFAP, NFL, and P-tau181 were quantified using an ultra-sensitive Simoa technology (Quanterix, MA, United States) on the automated Simoa HD-X platform (GBIO, Hangzhou, China), according to the manufacturer’s instruction. The Neurology 4-Plex E Assay Kit (Cat No:103670) and Ptau181 Advantage V2 Assay Kit (Cat No:103714) were purchased from Quanterix and used accordingly. Plasma samples were diluted at a 1:4 ratio for measurement. Calibrators, internal quality controls, and all samples were measured in duplicate. The mean coefficients of variation (CVs) of duplicate measurement for concentration were 2.83% (Aβ40), 3.31% (Aβ42), 4.48% (GFAP), 3.22% (NFL), and 5.81% (P-tau181). Few samples with intra-assay CVs larger than 20% were re-measured. The values were discarded if the variance was still >20% after being re-measured. The assays were performed using kits with the same lot number. Operators were unaware of the participants’ disease status.

### 2.5. β-Amyloid PET scan procedure and visual reading

Brain images were acquired with the patient in the supine position using a dedicated PET/CT scanner (PoleStar m660; SinoUnion Healthcare Inc., Beijing, China). The brain low-dose CT scan (120 kV, 260 mAs, 2.5 mm layer thickness, and 512 × 512 matrix) and PET scan (512 × 512 matrix) were obtained 45 min after the intravenous injection of 307–470 MBq (8.3–12.7 mCi) of ^18^F-AV45 which was synthesized in the cyclotron facility of our institute. The PET scan duration is 20 min. The emission data were corrected for scattering and attenuation. The PET images were reconstructed using ordered subsets expectation maximization (OSEM: 10 subsets, 4 iterations, and FWHM of 2.5 mm) with the time-of-flight (TOF) technique. The PET/CT images were reviewed by three specialists in nuclear medicine who were blinded to the MRI and clinical data. Three experienced nuclear medicine physicians visually analyze PET images to assess the radioactive distribution of the cerebral cortex. The scans were rated as positive or negative for the presence of Aβ pathology.

### 2.6. *APOE* genotyping

*APOE* genotype was determined according to previous research (Dong et al., 2021). DNA was extracted from white blood cells. *APOE* genotyping was obtained by sequencing the codons 112 and 158 of exon 4 of the *APOE* gene. The results are classified as *APOE ε4* non-carriers (*APOE ε4−/−*), heterozygotes (*APOE ε4+/−*), and homozygotes (*APOE ε4+/+*).

### 2.7. Clinical diagnostic criteria and CSF biomarkers diagnostic criteria

The clinical diagnostic criteria for patients are described later. The clinical diagnosis referred to the 2011 NIA-AA criteria for AD (McKhann et al., 2011), the Dementia with Lewy bodies (DLB) Consortium consensus for probable DLB (McKeith et al., 2017), the 2007 consensus criteria for Parkinson’s disease dementia (PDD; Emre et al., 2007), the 2011 Rascovsky criteria for behavioral variant frontotemporal dementia (bvFTD; Rascovsky et al., 2011), the 2011 Gorno-Tempini recommendation for primary progressive aphasia (PPA; Gorno-Tempini et al., 2011), the 2017 Hoglinger criteria for PSP (Hoglinger et al., 2017), the 2013 Armstrong’s criteria for CBS (Armstrong et al., 2013), the 1993 Report of the NINDS-AIREN International Workshop for VaD (Roman et al., 1993), and the Reilmann criteria for Huntington’s disease (HTD; Reilmann et al., 2014). Neuronal intranuclear inclusion disease (NIID) was diagnosed based on clinical history, imaging, NOTCH2NLC gene, and/or skin biopsy because of the lack of diagnostic criteria (Sone et al., 2016).

Based on the biomarker diagnostic criteria, the participants were divided into two subgroups: 1. Alzheimer’s continuum (Jack et al., 2018): CSF T-tau/Aβ42 > 0.5 or β-amyloid PET positive, 2. non-AD: CSF T-tau/Aβ42 ≤ 0.5 and β-amyloid PET negative. Furthermore, among the Alzheimer’s continuum, participants’ CSF P-tau181 levels of >50 pg./mL were defined as AD. These cutoff values were defined by our laboratory.

### 2.8. Statistical analysis

The statistical analyses were performed using SPSS 23.0. Data were expressed as mean ± standard deviation (SD). The Fisher exact *t*-test or *χ*^2^ test was used for categorical variables. The *t*-test and analysis of variance (*ANOVA*) were used for continuous variables. *ANOVA* was used for the comparison of multiple groups with the least significant difference (*LSD*) *post-hoc* test. Comparisons of CSF and plasma data were conducted using analysis of covariance (*ANCOVA*, covariates: age, sex, education, and disease duration), and Bonferroni tests were used for *post-hoc* comparisons. *p* < 0.05 was considered to be significant. All figures were produced with GraphPad Prism 8 software program.

## 3. Results

### 3.1. Demographics and biomarkers values

Among the 297 individuals, 52.2% (155/297) were women. The average disease duration was 3.4 ± 2.4 years. The average age was 61.5 ± 8.5 years. In total, 32.7% (97/297) of patients had a family history of dementia. The average educational level was 10.3 ± 4.2 years.

According to clinical diagnostic criteria, there were 174 cases with AD, 56 cases with FTLD, 31 cases with VaD, 18 cases with LBD, 14 cases with mixed dementia (AD-VaD), 2 cases with NIID, and 2 cases with HTD.

Based on the biomarker diagnostic criteria, 169 participants were diagnosed with Alzheimer’s continuum and 128 individuals were diagnosed with non-AD. Of the 169 patients with Alzheimer’s continuum, 120 patients were diagnosed with AD.

The following report was based on the biomarker diagnosis.

Table 1 shows the characteristics and biomarker values per group. Compared with non-AD patients, the AD continuum and AD patients showed a lower proportion of *APOE* ε4 non-carriers and a higher proportion of *APOE* ε4/ε4 genotype (*p* < 0.001). Furthermore, AD and AD continuum subjects exhibited lower MMSE scores than non-AD patients (*p* < 0.001; *p* < 0.001). There were no significant differences between AD continuum/AD and non-AD in age, gender, disease duration, educational level, and family history of dementia.

**Table 1:** Demographics, genetic data, and fluid biomarkers.

	All (297)	AD (120)	AD continuum (169)	non-AD (128)	P1*	P2*
Age, years	61.5 ± 8.5	60.8 ± 7.9	61.3 ± 8.0	61.7 ± 9.1	0.379	0.673
Female (%)	155 (52.2)	67 (55.8)	95 (56.2)	60 (46.9)	0.158	0.111
Disease duration, years	3.4 ± 2.4	3.4 ± 2.2	3.3 ± 2.2	3.5 ± 2.7	0.696	0.494
Education, years	10.3 ± 4.2	10.3 ± 4.4	10.4 ± 4.1	10.1 ± 4.4	0.747	0.638
Family history of dementia (%)	97.0 (32.7)	41.0 (13.8)	56.0 (33.1)	41 (32.0)	0.721	0.841
MMSE	13.6 ± 8.3	11.1 ± 7.3	11.8 ± 7.3	15.9 ± 9.0	**<0.001**	**<0.001**
APOE Genetic						
ε4ε4, *n* (%)	21 (7.1)	17 (14.2)	20 (11.8)	1 (0.8)	**<0.001**	**<0.001**
ε3ε4, *n* (%)	83 (27.9)	34 (28.3)	53 (31.4)	30 (23.4)		
ε3ε3, *n* (%)	169 (56.9)	64 (53.3)	87 (51.5)	82 (64.1)		
ε2ε3, *n* (%)	21 (7.1)	3 (2.5)	7 (4.1)	14 (10.9)		
ε2ε4, *n* (%)	2 (0.7)	2 (1.7)	2 (1.2)	0		
ε2ε2, *n* (%)	1 (0.3)	0	0	1 (0.8)		
ε4−/−, *n* (%)	191 (64.3)	67 (55.8)	94 (55.6)	97 (75.8)	**<0.001**	**<0.001**
ε4+/−, *n* (%)	85 (28.6)	36 (30.0)	55 (32.5)	30 (23.4)		
ε4+/+, *n* (%)	21 (7.1)	17 (14.2)	20 (11.8)	1 (0.8)		
CSF biomarkers						
Aβ42 (pg/mL)	575.5 ± 238.5	478.3 ± 134.4	475.9 ± 139.9	707.1 ± 275.7	**<0.001**	**<0.001**
T-tau (pg/mL)	381.9 ± 355.0	616.6 ± 407.2	543.9 ± 390.0	168.0 ± 107.0	**<0.001**	**<0.001**
P-tau181 (pg/mL)	57.5 ± 32.5	84.7 ± 31.0	70.6 ± 34.5	40.2 ± 18.8	**<0.001**	**<0.001**
T-tau/Aβ42	0.79 ± 0.87	1.36 ± 0.96	1.21 ± 0.97	0.25 ± 0.13	**<0.001**	**<0.001**
P-tau181/Aβ42	0.12 ± 0.09	0.19 ± 0.09	0.16 ± 0.09	0.06 ± 0.03	**<0.001**	**<0.001**
Plasma biomarkers						
Aβ40 (pg/mL)	76.5 ± 26.5	80.5 ± 28.6	77.6 ± 26.8	73.5 ± 25.8	0.088	0.255
Aβ42 (pg/mL)	5.3 ± 1.8	5.3 ± 1.7	5.1 ± 1.6	5.7 ± 2.1	0.544	0.173
P-tau181	4.1 ± 2.2	4.9 ± 1.9	4.8 ± 2.2	2.7 ± 1.4	**<0.001**	**<0.001**
GFAP (pg/mL)	166.6 ± 90.6	198.8 ± 79.4	190.4 ± 90.4	107.1 ± 58.4	**<0.001**	**<0.001**
NFL (pg/mL)	31.0 ± 34.9	27.4 ± 29.7	26.8 ± 30.0	41.4 ± 43.6	0.193	0.095
Aβ42/Aβ40	0.07 ± 0.02	0.06 ± 0.03	0.06 ± 0.02	0.07 ± 0.02	**0.020**	**0.010**
P-tau181/Aβ42	0.89 ± 0.58	1.01 ± 0.47	1.01 ± 0.53	0.57 ± 0.59	**<0.001**	**<0.001**

Aβ42, β amyloid 42; ADL, activities of daily living; APOE, apolipoprotein-E; CSF, cerebrospinal fluid; GFAP, glial fibrillary acidic protein; MMSE, mini-mental state examination; NFL, neurofilament light chain; P-tau, phosphorylated tau; T-tau, total tau. P1, AD vs. non-AD; P2, AD continuum vs. non-AD. ^*^p-values were computed by analysis of covariance, adjusting for age, sex, education, disease duration, and APOE genotype. *p*-values below 0.05 are bolded.

The Alzheimer’s continuum group and AD group showed lower CSF levels of Aβ42 (*p* < 0.001, *p* < 0.001) and higher CSF levels of T-tau (*p* < 0.001, *p* < 0.001), P-tau181 (*p* < 0.001, *p* < 0.001), T-tau/Aβ42 ratios (*p* < 0.001, *p* < 0.001), and P-tau181/Aβ42 ratios (*p* < 0.001, *p* < 0.001) than non-AD (Table 1).

Compared with non-AD patients, AD continuum and AD participants showed increased levels of plasma P-tau181 (*p* < 0.001, *p* < 0.001), GFAP (*p* < 0.001, *p* < 0.001) and P-tau181/Aβ42 (*p* < 0.001, *p* < 0.001) and decreased levels of Aβ42/Aβ40 ratios (*p* = 0.020, *p* = 0.010). However, the plasma levels of Aβ42, Aβ40, and NFL did not reach statistical significance between AD continuum/AD and non-AD (Table 1).

### 3.2. CSF biomarkers and *APOE ε4*

**Figure 1:**
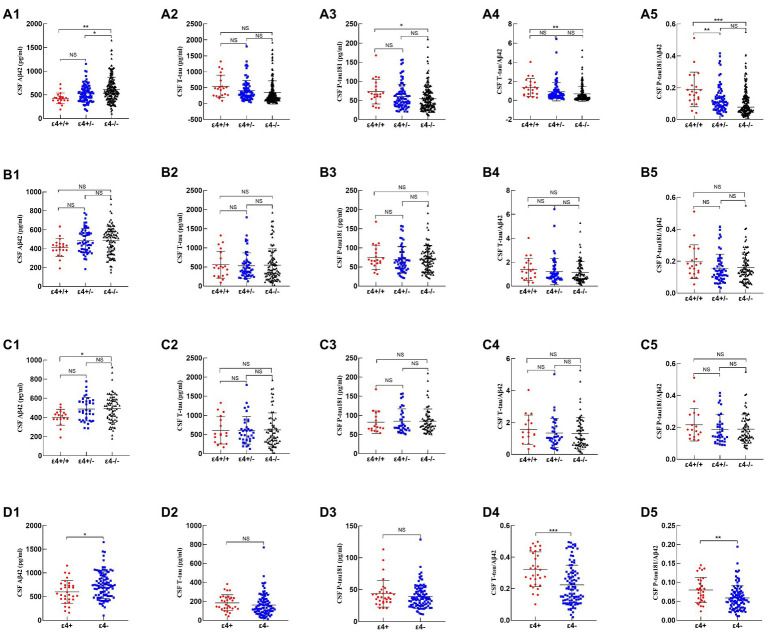
Comparison of CSF biomarkers among APOE genotypes. 1–5 represented CSF Aβ42, T-tau, P-tau181, T-tau/Aβ42, and P-tau181/Aβ42, respectively. (a1–a5), (b1–b5), (c1–c5), and (d1–d5) represented CSF biomarkers of all subjects, such as Alzheimer’s continuum, AD, and non-AD, respectively. *p*-values were calculated and were shown above the boxes as stars (***p < 0.001, **p-value < 0.01, *p-value < 0.05, “NS”, and not significant *p* > 0.05).

In the total cohort, CSF Aβ42 was lower in *APOE ε4/ε4* carriers (*p* = 0.001) and *APOE ε4* heterozygous carriers (*p* = 0.012) than in non-carriers. In addition, CSF P-tau181 (*p* = 0.027), T-tau/Aβ42 (*p* = 0.002), and P-tau181/Aβ42 (*p* < 0.001) were higher in *APOE ε4/ε4* carriers compared to *APOE ε4* non-carriers (Figures 1a1–a5; Supplementary Table).

Among Alzheimer’s continuum participants, the CSF biomarkers did not differ by *APOE ε4* status (Figures 1b1–b5; Supplementary Table). Among the AD patients, only CSF Aβ42 was lower in *APOE ε4/ε4* carriers than in non-carriers (*p* = 0.024; Figures 1c1–c5; Supplementary Table 1).

Among the non-AD patients, *APOE ε4* carriers showed lower CSF Aβ42 (*p* = 0.018), higher T-tau/Aβ42 (*p* < 0.001), and higher P-tau181/Aβ42 (*p* = 0.002) relative to *APOE ε4* non-carriers (Figures 1d1–d5; Supplementary Table).

### 3.3. Plasma biomarkers and *APOE ε4*

As shown in Table 2, the *APOE ε4* allele was not associated with plasma Aβ42, Aβ40, P-tau181, GFAP, and NFL levels and Aβ42/Aβ40 and P-tau181/Aβ42 ratios in the total cohort. Similarly, the *APOE ε4* did not affect the plasma biomarkers among Alzheimer’s continuum, AD, or non-AD patients (Table 2).

**Table 2:** Comparison of plasma biomarker levels of all patients, Alzheimer’s continuum, AD, and non-AD among the different ApoE ε4 genotypes.

	ε4+/+	ε4+/−	ε4−/−	Value of p*
**All (*n*)**	13	40	91	
Age, years	64.4 ± 8.1	63.6 ± 8.2	61.3 ± 7.3	0.173^$^
Femal (%)	8 (61.5)	21 (52.5)	47 (51.6)	0.799
Disease duration, years	3.0 ± 2.1	3.6 ± 2.6	3.4 ± 2.2	0.692^$^
Education, years	10.1 ± 5.1	9.9 ± 4.1	9.6 ± 4.2	0.886^$^
MMSE	11.9 ± 7.9	12.2 ± 7.5	12.8 ± 7.6	0.882^$^
Aβ40 (pg/mL)	77.0 ± 17.5	74.0 ± 22.5	77.5 ± 29.2	0.643^*^
Aβ42 (pg/mL)	4.8 ± 1.1	4.9 ± 1.4	5.5 ± 2.0	0.116^*^
P-tau181	5.0 ± 1.5	4.1 ± 1.6	4.1 ± 2.5	0.163^*^
GFAP (pg/mL)	199.8 ± 97.7	155.8 ± 73.4	166.4 ± 95.8	0.307^*^
NFL (pg/mL)	22.6 ± 9.7	23.9 ± 27.4	35.2 ± 39.4	0.126^*^
Aβ42/Aβ40	16.17 ± 2.11	15.35 ± 2.05	15.25 ± 4.70	0.699^*^
P-tau181/Aβ42	1.08 ± 0.34	0.91 ± 0.56	0.85 ± 0.62	0.216^*^
**AD continuum (*n*)**	12	32	58	
Age, years	63.8 ± 8.2	63.3 ± 8.8	60.0 ± 7.1	0.091^$^
Femal (%)	7 (58.3)	16 (50.0)	31 (53.4)	0.879
Disease duration, years	3.1 ± 2.2	4.0 ± 2.6	2.9 ± 1.8	0.069^$^
Education, years	10.8 ± 4.4	9.8 ± 3.9	9.8 ± 4.8	0.746^$^
MMSE	11.9 ± 8.2	11.2 ± 7.2	11.9 ± 7.2	0.906^$^
Aβ40 (pg/mL)	78.8 ± 16.9	73.8 ± 23.1	79.3 ± 30.2	0.551^*^
Aβ42 (pg/mL)	4.8 ± 1.1	4.8 ± 1.4	5.3 ± 1.8	0.340^*^
P-tau181	5.0 ± 1.5	4.5 ± 1.4	4.9 ± 2.6	0.720^*^
GFAP (pg/mL)	200.5 ± 102.0	176.9 ± 66.8	195.3 ± 98.9	0.630^*^
NFL (pg/mL)	22.6 ± 10.1	23.8 ± 30.3	29.2 ± 32.6	0.207^*^
Aβ42/Aβ40	0.06 ± 0.01	0.06 ± 0.01	0.07 ± 0.03	0.860^*^
P-tau181/Aβ42	1.07 ± 0.35	1.04 ± 0.55	0.99 ± 0.56	0.786^*^
**AD (*n*)**	10	18	40	
Age, years	66.6 ± 5.5	61.5 ± 8.9	59.6 ± 7.5	**0.040** ^**$**^
Femal (%)	5 (50.0)	10 (55.6)	23 (57.5)	0.912
Disease duration, years	3.4 ± 2.4	4.6 ± 2.8	2.7 ± 1.9	**0.013** ^**$**^
Education, years	11.9 ± 4.0	8.9 ± 4.5	9.7 ± 5.2	0.288^$^
MMSE	12.3 ± 8.4	9.1 ± 6.3	11.0 ± 7.2	0.471^$^
Aβ40 (pg/mL)	83.8 ± 11.5	75.2 ± 25.5	82.0 ± 32.7	0.960^*^
Aβ42 (pg/mL)	5.1 ± 0.9	4.7 ± 1.5	5.6 ± 1.8	0.346^*^
P-tau181	5.2 ± 1.6	4.8 ± 1.6	4.9 ± 2.1	0.672^*^
GFAP (pg/mL)	214.2 ± 106.1	181.9 ± 50.8	202.2 ± 82.5	0.372^*^
NFL (pg/mL)	24.6 ± 9.9	26.0 ± 39.5	28.6 ± 28.7	0.391^*^
Aβ42/Aβ40	0.06 ± 0.01	0.06 ± 0.01	0.07 ± 0.03	0.977^*^
P-tau181/Aβ42	1.04 ± 0.35	1.15 ± 0.61	0.94 ± 0.42	0.609^*^
**non-AD**	**ε4+ (9)**		**ε4- (33)**	Value of p*
Age, years	65.3 ± 5.7		63.6 ± 7.1	0.511^$^
Femal (%)	6 (66.7)		16 (48.5)	0.333
Disease duration, years	2.2 ± 1.7		4.2 ± 2.6	**0.035** ^**$**^
Education, years	9.1 ± 5.6		9.3 ± 3.2	0.924^$^
MMSE	15.7 ± 7.3		14.3 ± 8.1	0.643^$^
Aβ40 (pg/mL)	72.3 ± 21.4		73.9 ± 27.3	0.947^*^
Aβ42 (pg/mL)	5.2 ± 1.5		5.8 ± 2.2	0.724^*^
P-tau181	2.7 ± 1.6		2.7 ± 1.4	0.906^*^
GFAP (pg/mL)	89.6 ± 47.9		112.1 ± 60.8	0.564^*^
NFL (pg/mL)	24.1 ± 12.3		46.4 ± 48.2	0.256^*^
Aβ42/Aβ40	0.07 ± 0.01		0.07 ± 0.02	0.355^*^
P-tau181/Aβ42	0.54 ± 0.35		0.58 ± 0.66	0.970^*^

Aβ42, β amyloid 42; ADL, activities of daily living; APOE, apolipoprotein-E; CSF, cerebrospinal fluid; GFAP, glial fibrillary acidic protein; MMSE, mini-mental state examination; NFL, neurofilament light chain; P-tau, phosphorylated tau; T-tau, total tau. Non-AD was divided into APOE ɛ4 carriers and non-carriers due to the limited number of patients carrying APOE ɛ4+/+. $, ANOVA was used for the comparison of multiple groups. *p-values were computed by analysis of covariance, adjusting for age, sex, education, and disease duration. *p*-values below 0.05 are bolded.

## 4. Discussion

In the present study, we confirmed that the *APOE ε4* allele was more prevalent in AD and Alzheimer’s continuum than in non-AD. Furthermore, the *APOE ε4/ε4* carriers accounted for 11.8% of Alzheimer’s continuum and 14.2% of biomarker-confirmed AD, which were higher than those previously reported in studies based on only clinical AD criteria (Ward et al., 2012; Yamazaki et al., 2019). A recent study also reported that *APOE ε4/ε4* accounted for 16.6% of biomarker-diagnosed AD (Saddiki et al., 2020). In addition, it argued that the biomarker diagnosis strengthened the association between AD and *APOE ɛ4* (Saddiki et al., 2020).

Plasma biomarkers for AD and other dementias are now becoming a reality. In AD patients, plasma biomarkers are abnormal in parallel with CSF biomarker values and thus can be a powerful tool for early and accurate diagnosis in clinical practice (Teunissen et al., 2022). We found that plasma concentrations of P-tau181, Aβ42/40 ratios, and P-tau181/Aβ42 ratios were significantly higher in the AD continuum and AD than in non-AD patients, which was similar to previous studies (Schindler et al., 2019; Janelidze et al., 2020; Thijssen et al., 2020; Li et al., 2022). Consistent with previous studies, our study also found that plasma GFAP levels were higher in AD patients than in non-AD (Benedet et al., 2021;Simren et al., 2021; Teunissen et al., 2022). GFAP was an astrocytic damage marker. Recent studies have described increased levels of GFAP in AD (Simren et al., 2021; Teunissen et al., 2022). Emerging evidence has shown reactive astrocytosis had been implicated as a potential driver or effect of AD pathological changes, and the elevated plasma GFAP levels were associated with amyloid pathology (Pereira et al., 2021; Teunissen et al., 2022). As for NFL, poor diagnostic performance has been reported for the separation of AD dementia from those with non-AD disorders, which was similar to existing results (Illan-Gala et al., 2021; Leuzy et al., 2022). Therefore, our findings supported the concept that plasma GFAP, P-tau181, Aβ42/40 ratios, and P-tau181/Aβ42 were promising biomarkers for AD.

We also confirmed that in patients with AD, there were decreased CSF levels of Aβ42 in *APOE ε4/ε4* carriers, which is in agreement with previous studies (Lautner et al., 2014; Vogelgsang et al., 2019). The potential mechanisms underlying the association between *APOE ε4* and CSF levels of Aβ42 were not fully understood but may be partly related to the reduction of Aβ clearance and promotion of Aβ aggregation by *ε4* allele, thereby reducing CSF Aβ42 levels in *APOE ε4* carriers (Baek et al., 2020; Koutsodendris et al., 2022). *APOE ε4/ε4* only affected the level of CSF Aβ42 and did not affect the more diagnostic value of the T-tau/Aβ42 ratio. However, since *APOE ε4/ε4* was more prevalent in biomarker-diagnosed AD, it suggested that *APOE ε4/ε4* was closely related to the development of AD but had no further influence on the biomarkers after AD development.

Interestingly, in non-AD patients, a significant difference was found in levels of Aβ42 between *APOE ε4* carriers and non-carriers. A plausible explanation for this observation is the presence of some accompanying AD pathology in some non-AD subjects (Safieh et al., 2019). *APOE ε4* might exert effects on AD pathology. Previous studies have found that typical LBD was associated with increased occipital Aβ deposition through its interaction with *APOE ε4* (Jung et al., 2021). Furthermore, Aβ deposition was common in patients with LBD at autopsy (Kantarci et al., 2020). In addition, *APOE ε4* may influence Aβ deposition in CAA by affecting Aβ clearance and aggregation, and patients with CAA have reduced CSF levels of Aβ42 (Yamada, 2015; Belloy et al., 2019). At present, no study had found the effect of *APOE ε4* on Aβ metabolism in VaD, FTLD, NIID, and HTD. However, *APOE* had been found to be a risk factor for VAD and FTD (Rohn, 2014; Perry et al., 2017). The association of *APOE ε4* with the pathology the pathology of non-AD dementias could be further evaluated.

In addition, we did not find any associations of *APOE ε4/ε4* with plasma Aβ40, Aβ42, and their ratios. Our data were in agreement with the previous study demonstrating plasma levels of Aβ40 and Aβ42, and their ratios were not lower in *APOE ε4/ε4* carriers (Olsson et al., 2016). It was hypothesized that other factors may be regulating the peripheral levels of Aβ, including the production of plasma Aβ from the periphery, and that Aβ entering peripheral blood may be degraded by circulating enzymes or metabolized in the liver or bound to peripheral blood proteins (Roher et al., 2009).

In the present study, CSF levels of T-tau and P-tau181 were not influenced by *APOE ε4* in AD and non-AD. Similarly, plasma levels of P-tau181 were not different in *APOE ɛ4* carriers and non-carriers. These were consistent with the previous research (Lautner et al., 2014; Benson et al., 2022; Salami et al., 2022). Furthermore, we found that *APOE ɛ4* was not associated with plasma GFAP and NFL among AD or non-AD subjects. Few studies have explored the associations of *APOE ε4* with plasma levels of GFAP and NFL. Perhaps the effect of *APOE ɛ4* on AD pathology lay mainly in Aβ but not in tau, GFAP, and NFL.

The main limitation of this study was the small sample size. We found associations between *APOE ε4* and CSF AD core biomarkers in non-AD patients. Due to the limited sample size, we did not perform further detailed analysis in different non-AD types.

## 5. Conclusion

In conclusion, our data verified that of the three groups (AD continuum, AD, and non-AD), those with AD had the highest frequency of APOE ɛ4/ɛ4 genotypes. In addition, the *APOE ɛ4/ɛ4* was associated with the levels of CSF Aβ42 for AD and non-AD, suggesting that *APOE ɛ4/ɛ4* affected the Aβ metabolism of both. *APOE ε4/ɛ4* had no associations with plasma biomarkers in AD and non-AD.

## Data availability statement

The raw data supporting the conclusions of this article will be made available by the authors, without undue reservation.

## Ethics statement

The studies involving human participants were reviewed and approved by PUMCH ethics committees, Peking Union Medical College Hospital. The patients/participants provided their written informed consent to participate in this study.

## Author contributions

LS was involved in study design, acquisition, statistical analysis, and drafting and revising the manuscript. LD was involved in drafting and revising the manuscript. XH, TW, JL, and JW were involved in the acquisition and statistical analysis. CM and CL were involved in the study design. JG was involved in the study design and revision. All authors contributed to the manuscript revision and read and approved the submitted version.

## Funding

This study was financially supported by the National Key Research and Development Program of China (nos. 2020YFA0804500 and 2020YFA0804501), the CAMS Innovation fund for medical sciences (CIFMS) (nos. 2021-I2M-1-020 and 2020-I2M-C&T-B-010), the National Natural Science Foundation of China (nos. 81550021 and 30470618), and the Science Innovation 2030-Brain Science and Brain-Inspired Intelligence Technology Major Project (no. 2021ZD0201106).

## Conflict of interest

The authors declare that the research was conducted in the absence of any commercial or financial relationships that could be construed as a potential conflict of interest.

## Publisher’s note

All claims expressed in this article are solely those of the authors and do not necessarily represent those of their affiliated organizations, or those of the publisher, the editors and the reviewers. Any product that may be evaluated in this article, or claim that may be made by its manufacturer, is not guaranteed or endorsed by the publisher.

## Figures and Tables

**Table:** 

AD	Alzheimer’s disease
ADL	activities of daily living
*APOE*	*apolipoprotein-E*
bvFTD	behavioral variant frontotemporal dementia
CBS	corticobasal syndrome
CSF	cerebrospinal fluid
DLB	dementia with Lewy bodies
FTLD	frontotemporal lobar degeneration
GFAP	glial fibrillary acidic protein
HAD	hospital anxiety and depression scale
HTD	Huntington’s disease
LBD	Lewy body disease
MMSE	mini-mental state examination
NFL	neurofilament light chain
NIA-AA	National Institute on Aging Alzheimer’s Association
NIID	neuronal intranuclear inclusion disease
PDD	Parkinson’s disease dementia
PPA	primary progressive aphasia
PUMCH	Peking Union Medical College Hospital
SD	standard deviation
VAD	vascular dementia
