# Supplementary material for: Association of APOE ε4/ε4 with fluid biomarkers in patients from the PUMCH dementia cohort
Source: Front Aging Neurosci. 2023 Mar 31;15:1119070. doi: 10.3389/fnagi.2023.1119070 (PMC10103647; doi:10.3389/fnagi.2023.1119070)
Supplement: Supplementary file 1 [file Table_1.DOCX]

Supplementary Material

Influence of APOE ε4/ε4 on Fluid Biomarkers in Patients from the PUMCH Dementia Cohort

Li Shang, Liling Dong*†, Xinying Huang, Tianyi Wang, Chenhui Mao, Jie Li, Jie Wang1, Caiyan Liu, Jing Gao*†

*** Correspondence:** (1) Jing Gao: [gj107@163.com;](mailto:gj107@163.com;) (2) Liling Dong: sophie_d@163.com

†These authors contributed equally to this work and share corresponding author

# Supplementary Table

| **Supplement Table. Comparison of CSF biomarker levels of all patients, Alzheimer's continuum, AD and non-AD among the different ApoE ε4 genotypes** | | | | | | | |
| --- | --- | --- | --- | --- | --- | --- | --- |
|  | **ε4+/+** | **ε4+/-** | **ε4-/-** | ***P*** | ***P1*** | ***P2*** | ***P3*** |
| **All (n)** | 21 | 85 | 191 |  |  |  |  |
| Age, years | 64.0±8.5 | 61.6±9.1 | 61.2±8.2 | 0.373^$^ | 0.255 | 0.161 | 0.730 |
| Femal (%) | 12 (57.1) | 50 (58.8) | 93 (48.7) | 0.267 |  |  |  |
| Disease duration, years | 3.1±2.0 | 3.5±2.3 | 3.4±2.5 | 0.831^$^ | 0.545 | 0.629 | 0.780 |
| Education, years | 10.7±4.5 | 10.8±4.0 | 10.0±4.3 | 0.257^$^ | 0.874 | 0.465 | 0.114 |
| Familary history of dementia (%) | 6 (28.6) | 32 (37.6) | 59 (30.9) | 0.498 |  |  |  |
| MMSE | 11.6±8.0 | 13.9±8.2 | 13.5±8.4 | 0.511^$^ | 0.248 | 0.304 | 0.730 |
| Aβ42 (pg/ml) | 427.0±114.2 | 524.0±183.2 | 614.8±259.2 | **<0.001^*^** | 0.229 | **0.001** | **0.012** |
| T-tau (pg/ml) | 543.7±352.5 | 408.7±321.2 | 352.1±365.4 | **0.049^*^** | 0.293 | 0.054 | 0.826 |
| P-tau181 (pg/ml) | 73.2±32.4 | 60.1±32.5 | 54.6±32.0 | **0.025^*^** | 0.202 | **0.027** | 0.673 |
| T-tau/Aβ42 | 1.35±0.93 | 0.90±0.97 | 0.68±0.79 | **0.001^*^** | 0.078 | **0.002** | 0.165 |
| P-tau181/Aβ42 | 0.19±0.11 | 0.13±0.08 | 0.11±0.08 | **<0.001^*^** | **0.006** | **<0.001** | 0.278 |
| **AD continuum (n)** | 20 | 55 | 94 |  |  |  |  |
| Age, years | 63.6±8.6 | 61.8±8.9 | 60.6±7.3 | 0.266^$^ | 0.395 | 0.126 | 0.358 |
| Femal (%) | 11 (55.0) | 32 (58.2) | 52 (55.3) | 0.937 |  |  |  |
| Disease duration, years | 3.2±2.0 | 3.7±2.5 | 3.1±2.0 | 0.273^$^ | 0.359 | 0.901 | 0.114 |
| Education, years | 11.2±4.0 | 10.6±3.9 | 10.1±4.3 | 0.499^$^ | 0.598 | 0.284 | 0.456 |
| Familary history of dementia (%) | 6 (30.0) | 20 (36.4) | 30 (31.9) | 0.814 |  |  |  |
| MMSE | 11.6±8.1 | 12.5±7.4 | 11.3±7.0 | 0.626^$^ | 0.608 | 0.907 | 0.339 |
| Aβ42 (pg/ml) | 412.0±93.7 | 485.0±128.6 | 484.2±151.6 | 0.069^*^ | 0.102 | 0.078 | 1.000 |
| T-tau (pg/ml) | 558.9±354.5 | 531.4±336.4 | 547.9±428.1 | 0.920^*^ | 1.000 | 1.000 | 1.000 |
| P-tau181 (pg/ml) | 75.4±31.6 | 68.9±34.4 | 70.6±35.3 | 0.595^*^ | 0.926 | 1.000 | 1.000 |
| T-tau/Aβ42 | 1.40±0.93 | 1.22±1.08 | 1.16±0.91 | 0.506^*^ | 1.000 | 0.733 | 1.000 |
| P-tau181/Aβ42 | 0.20±0.10 | 0.15±0.09 | 0.16±0.09 | 0.112^*^ | 0.118 | 0.206 | 1.000 |
| **AD (n)** | 17 | 36 | 67 |  |  |  |  |
| Age, years | 64.1±6.9 | 60.3±9.0 | 60.2±7.4 | 0.184^$^ | 0.105 | 0.075 | 0.974 |
| Femal (%) | 9 (52.9) | 22 (61.1) | 36 (53.7) | 0.747 |  |  |  |
| Disease duration, years | 3.4±2.1 | 3.9±2.6 | 3.1±2.1 | 0.203^$^ | 0.423 | 0.621 | 0.075 |
| Education, years | 11.7±3.7 | 10.3±4.6 | 10.0±4.4 | 0.344^$^ | 0.270 | 0.145 | 0.728 |
| Familary history of dementia (%) | 6 (35.3) | 14 (38.9) | 21 (31.3) | 0.739 |  |  |  |
| MMSE | 11.8±8.5 | 11.5±7.2 | 10.8±7.1 | 0.829^$^ | 0.871 | 0.604 | 0.653 |
| Aβ42 (pg/ml) | 402.5±81.7 | 487.5±122.6 | 492.6±145.7 | **0.022^*^** | 0.039 | **0.024** | 1.000 |
| T-tau (pg/ml) | 607.9±357.0 | 611.6±362.5 | 621.5±445.4 | 0.961^*^ | 1.000 | 1.000 | 1.000 |
| P-tau181 (pg/ml) | 82.3±29.1 | 85.2±31.6 | 85.0±31.6 | 0.939^*^ | 1.000 | 1.000 | 1.000 |
| T-tau/Aβ42 | 1.55±0.92 | 1.34±0.92 | 1.32±0.99 | 0.480^*^ | 0.787 | 0.769 | 1.000 |
| P-tau181/Aβ42 | 0.22±0.10 | 0.19±0.09 | 0.19±0.09 | 0.320^*^ | 0.427 | 0.564 | 1.000 |
| **non-AD** | **ε4+ (31)** | | **ε4- (97)** | ***P*** |  |  |  |
| Age, years | 61.5±9.5 | | 61.8±9.1 | 0.857^#^ |  |  |  |
| Femal (%) | 19 (61.3) | | 41 (42.3) | 0.065 |  |  |  |
| Disease duration, years | 3.1±1.9 | | 3.7±2.9 | 0.262^#^ |  |  |  |
| Education, years | 11.0±4.5 | | 9.9±4.3 | 0.226^#^ |  |  |  |
| Familary history of dementia (%) | 12 (38.7) | | 29 (29.9) | 0.360 |  |  |  |
| MMSE | 16.3±9.0 | | 15.7±9.1 | 0.739^#^ |  |  |  |
| Aβ42 (pg/ml) | 599.8±238.5 | | 741.3±279.0 | **0.018^*^** |  |  |  |
| T-tau (pg/ml) | 185.4±83.3 | | 162.4±113.4 | 0.288^*^ |  |  |  |
| P-tau181 (pg/ml) | 43.6±20.8 | | 39.1±18.0 | 0.219^*^ |  |  |  |
| T-tau/Aβ42 | 0.32±0.11 | | 0.22±0.13 | **<0.001^*^** |  |  |  |
| P-tau181/Aβ42 | 0.08±0.03 | | 0.06±0.03 | **0.002^*^** |  |  |  |

Abbreviation: Aβ42, β amyloid 42; APOE, apolipoprotein-E; CSF, cerebrospinal fluid; P-tau，phosphorylated tau；T-tau, total tau. non-AD patients were divided into APOE ɛ4 carriers and non-carriers because of the limited number of patients carrying APOE ɛ4+/+. *,p Values were computed by analysis of covariance, adjusting for age, sex, education and disease duration. $,ANOVA was used for the comparison of multiple groups. #, Independent samples t-test. P1, P2, P3 were coputed by post-hoc comparisons. P1, ε4+/+ vs.ε4+/-; P2, ε4+/+ vs.ε4-/-; P3, ε4+/- vs.ε4-/-.

**
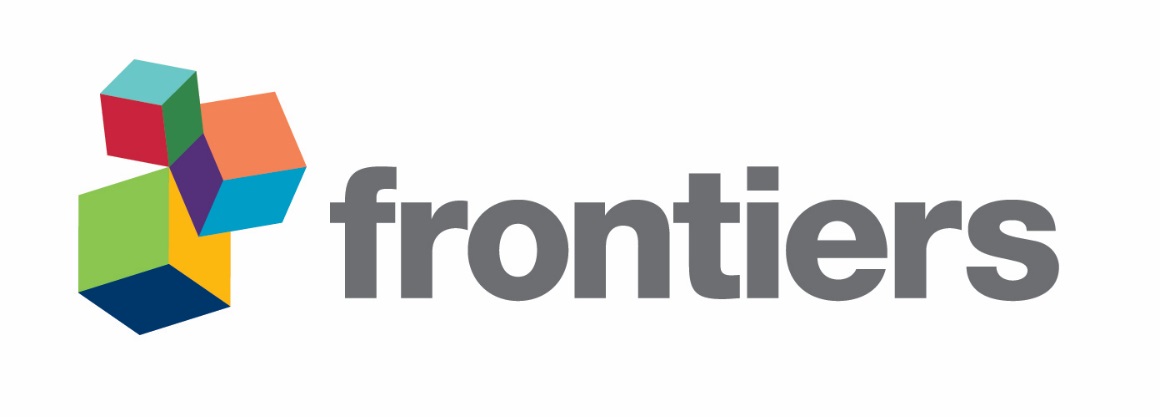
**
